# Evaluation externe de la qualité de la numération des lymphocytes T-CD4 au laboratoire d’immunologie et hématologie du CHU de Cocody

**DOI:** 10.11604/pamj.2018.30.200.11619

**Published:** 2018-07-06

**Authors:** Koffi N’guessan, Yekayo Benedicte Koné, Oppong Richard Yéboah, Amah Patricia Goran-Kouacou, Séry Romuald Dassé

**Affiliations:** 1 Université Félix Houphouët-Boigny d’Abidjan, Laboratoire d’Immunologie et Hématologie, Centre Hospitalier Universitaire de Cocody, Abidjan, Côte d’Ivoire

**Keywords:** CD4, EEQ, Côte d´Ivoire, QASI, CD4, EQAP, I vory Coast, QASI

## Abstract

**Introduction:**

Le taux de lymphocytes T-CD4 (LT4) est un paramètre crucial de suivi et de décision thérapeutique au cours de l'infection au Virus de l'Immunodéficience Humaine (VIH). Sa détermination doit être fiable et précise. Pour fournir des résultats de qualité, le laboratoire d'immunologie et hématologie du Centre Hospitalier et Universitaire (CHU) de Cocody participe à l'Evaluation Externe de la Qualité (EEQ) de la numération des LT4 du QASI *(Quality Assessment and Standardization for Immunological Measures relevant to HIV/AIDS)*. L'objectif de notre travail était d'évaluer la performance du laboratoire dans la numération des LT4.

**Méthodes:**

Une étude rétrospective a été menée sur les rapports de performance d'EEQ du laboratoire. Les indicateurs de performances SDI (indice de déviation standard), CV (coefficient de variation) ont été évalués.

**Résultats:**

Le taux de participation au programme était de 83,33%. Les indicateurs de performance étaient satisfaisants. Les SDI étaient en majorité inclus dans l'intervalle de confiance [-2; +2]. Les CV des échantillons de valeurs normales de CD4 étaient dans les normes. Les taux de conformité des résultats CD4 rendus étaient respectivement de 89,58% et 91,87% pour le compte absolu et le compte relatif. Seuls les CV des échantillons de faible taux de CD4 étaient au-delà des normes (> 15%).

**Conclusion:**

L'EEQ était un outil indispensable qui permettait au laboratoire de surveiller la qualité de ses analyses. Toutefois des mesures correctives devraient être renforcées dans le suivi-évaluation pour pérenniser et améliorer la qualité des analyses.

## Abstract (en)

**Introduction:**

The rate of T lymphocytes-CD4 (TLR4) is a crucial parameter for therapeutic decision and follow-up in patients with Human Immunodeficiency Virus (HIV) infection. This must be determined in a reliable and accurate way. The laboratory of immunology and hematology at the Cocody University Hospital Center has participated in the External Quality Assessment program (EQAP) for TLR4 enumeration provided by QASI (Quality Assessment and Standardization for Immunological Measures Relevant to HIV/AIDS) in order to deliver quality results. This study aims to assess laboratory performance in TLR4 enumeration.

**Methods:**

We conducted a retrospective study to evaluate the laboratory performance reports from EQAP. Performance indicators such as SDI (Standard Deviation Index) and CV (coefficient of variation) were evaluated.

**Results:**

Program participation rate was 83.33%. Performance indicators were satisfactory. The majority of SDI were included in the confidence interval [-2; +2]. The CV of samples with normal CD4 values were within the standards. Compliance rate of CD4 results was 89.58% and 91.87% for the absolute and the relative performance respectively. The CV of samples with low CD4 rates were beyond the standards (>15%).

**Conclusion:**

EQAP is an indispensable tool enabling laboratory to monitor the quality of its analyses. However, corrective measures should be strengthened during the follow-up period to sustain and improve the quality of the analyses.

## Introduction

La pandémie de l'infection par le Virus de l'Immunodéficience Humaine (VIH) représente un problème majeur de santé publique. Dans sa fiche d'information 2016 intitulée « statistiques mondiales », le programme commun des Nations Unies sur le VIH/SIDA (ONUSIDA) estimait à environ 36,7 millions le nombre de personnes vivant avec le VIH (PVVIH) dans le monde en 2015. L'Afrique subsaharienne reste toujours la région du monde la plus durement affectée avec 25,5 millions de personnes infectées [1]. Les lymphocytes T-CD4 (LT4) qui organisent la réponse immunitaire sont la cible principale du virus. Les LT4 sont détruits préférentiellement provoquant ainsi un déficit immunitaire à l'origine de nombreuses infections ou maladies opportunistes. La détermination du taux de LT4 est ainsi utilisée pour évaluer le degré de détérioration immunitaire et la vitesse de progression de la maladie vers le stade SIDA [2, 3]; pour améliorer la surveillance de la maladie [4]; pour déterminer le moment optimal pour la prophylaxie des infections opportunistes [5]; et pour surveiller l'efficacité du traitement antirétroviral [6]. Le taux de LT4 s'avère être ainsi un paramètre crucial de suivi et de décision thérapeutique au cours de l'infection au VIH, dont la détermination doit être fiable et d'une grande précision. Dans le souci de fournir des résultats précis et fiables, le laboratoire d'immunologie et hématologie du CHU (Centre Hospitalier Universitaire) de Cocody s'est inscrit dans un programme d'évaluation externe de la qualité (EEQ) assuré par le QASI *(Quality Assessment and Standardization for Immunological Measures relevant to HIV/AIDS)*. Le QASI est un programme international de l'agence de santé publique du Canada qui a été créé en 1996 pour répondre aux besoins des laboratoires de phénotypage immunologique en matière d'évaluation de la compétence dans les pays où ce service n'existe pas encore [7]. L'objectif de notre travail était d'évaluer la performance du laboratoire d'immunologie et hématologie pour la numération des LT4 des personnes vivant avec le VIH au cours de l'EEQ.

## Méthodes

**Type de l'étude**: Une étude rétrospective, descriptive et analytique a été réalisée au laboratoire d'immunologie et hématologie du CHU de Cocody à Abidjan (Côte d'Ivoire) au cours du 2^ème^ trimestre de l'année 2016.

**Matériel**: Les archives de l'unité PVVIH du laboratoire d'immunologie et d'hématologie ont été exploitées. Les données étudiées étaient essentiellement les rapports de performance établis par le QASI après l'envoi des résultats des analyses des échantillons d'EEQ. Ces échantillons étaient à chaque évaluation, deux tubes de sang stabilisé non infectieux de 1ml fourni par le QASI: un échantillon avec des valeurs faibles de CD4 et un autre avec des valeurs normales de CD4. Pour la numération des LT4, le laboratoire utilisait comme cytomètre, un appareil FacsCalibur ^®^ (Becton Dickinson, Californie, US).

**Paramètres évalués**: La régularité de la participation du laboratoire. Elle a été appréciée par le taux de participation du laboratoire à l'EEQ depuis l'inclusion au programme; l'indice de déviation standard (SDI), encore appelé indice d'écart-type du rapport de performance, calculé par le QASI. Cette donnée statistique évalue la performance des résultats de la numération des LT4. Classiquement, on considère que le résultat est “hors-limites” si SDI < -2 ou SDI > 2; la qualité globale des résultats. Il s'agit du pourcentage de résultats conformes par rapport au nombre total des résultats rendus. C'est un indicateur de qualité qui varie entre 0 (aucune valeur conforme) et 100% (tous les résultats sont conformes). Le laboratoire a d'autant mieux travaillé que son pourcentage de résultats conformes est élevé. Le nombre de résultats conformes correspond au nombre de SDI compris dans l'intervalle (-2; +2); la précision des résultats. Elle est appréciée par le coefficient de variation (CV). Les normes recommandent que le CV soit inférieur à 15%. CV=SD/M (SD: standard déviation; M: moyenne des valeurs du groupe de laboratoires participant à l'évaluation dont les SDI compris dans l'intervalle -2; +2). Les indicateurs (SDI; SD; M) étaient disponibles dans les rapports de performance fournis par le QASI.

## Résultats

Le cytomètre du laboratoire était un appareil FACSCalibur ^®^ qui permettait de faire une numération absolue des CD4 mais également de donner le pourcentage des CD4. La technique de numération au laboratoire se faisait en *lysing* simple plateforme et utilisait le panel CD3-FITC/CD4-PE/CD45-PerCP avec des tubes *TruCount*.

**Figure 1:**
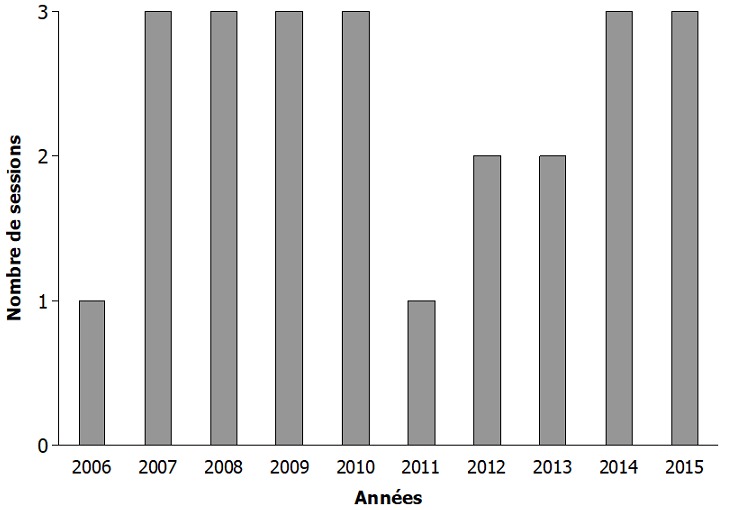
Représentation des participations du laboratoire aux sessions d’évaluation externe de la qualité de la numération des Lymphocytes T-CD4 par année

**Taux de participation du laboratoire à l'EEQ**: L'exploitation des archives a permis de noter que 3 sessions d'EEQ étaient réalisées chaque année par le QASI. Le laboratoire d'immunologie et hématologie du CHU de Cocody participait au programme d'EEQ depuis la dernière session de l'année 2006. Du début de l'inclusion jusqu'à fin 2015, 24 sessions d'évaluation avaient été réalisées par le QASI. Pour cette période, le laboratoire n'avait pas participé à toutes les sessions notamment 4 sessions, ce qui représentait un taux de participation au programme de 83,33% (Figure 1).

**Figure 2:**
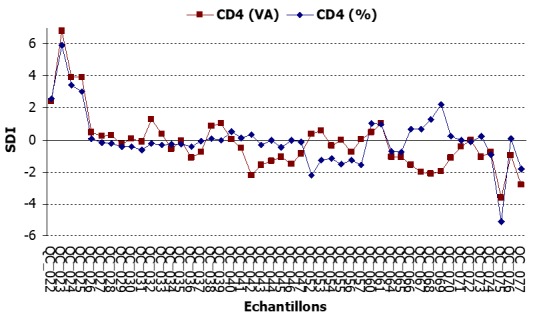
Évolution des valeurs de SDI du compte absolu et compte relatif de CD4

**Performance des résultats de la numération des LT4**: Les valeurs SDI, autant pour le compte absolu que le compte relatif étaient en majorité inclus dans l'intervalle de confiance (-2; +2) (Figure 2). Les SDI au début de l'inclusion au programme d'EEQ étaient en dehors de l'intervalle de confiance. Les SDI des sessions à partir des échantillons (QC_060, QC_061) oscillaient beaucoup plus dans l'intervalle de confiance et s'y écartaient par moment. Il n'y avait pas eu de rapport de performance pour les évaluations des numérations LT4 des échantillons (QC_048, QC_049); (QC_050, QC_051); (QC_058, QC_059); (QC_062, QC_063) qui correspondaient aux 4 sessions de non-participation.

**Figure 3:**
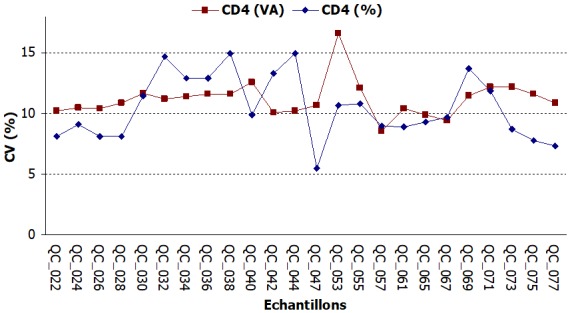
Évolution des valeurs de CV du compte absolu et compte relatif des valeurs normales de CD4

**Figure 4:**
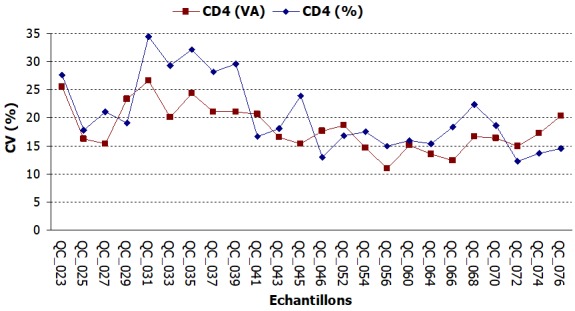
Évolution des valeurs de CV du compte absolu et du compte relatif des taux faibles de CD4

**Précision des résultats**: Les échantillons avec les valeurs normales de CD4 étaient ceux avec des numéros pairs de QC_022 à QC_045 et ceux avec les numéros impairs de QC_046 à QC_077 et le contraire pour les échantillons avec les valeurs faibles de CD4. Les valeurs de CV des échantillons avec des valeurs normales de CD4 oscillaient autour de 10% (Figure 3). Les CV des échantillons contenant un faible taux de CD4 étaient au-delà des normes (15%), elles oscillaient autour de 20% (Figure 4).

**Qualité globale des résultats**: Le pourcentage de résultats conformes durant la période de l'étude était de 89,58% pour le compte absolu de CD4 et de 91,87% pour le compte relatif de CD4.

## Discussion

En Côte d'Ivoire, la détermination du taux de CD4 est le marqueur biologique indispensable à réaliser pour l'initiation de la thérapie antirétrovirale puis tous les 6 mois pour le suivi thérapeutique. A cet effet, le laboratoire d'immunologie et hématologie du CHU de Cocody bénéficie de la part des partenaires de la lutte contre le VIH en Côte d'Ivoire, d'un FACSCalibur ^®^, de réactifs et de sessions de formation du personnel technique pour la numération des LT4. En plus des procédures de contrôle de qualité interne pour le comptage des CD4, le laboratoire participe à une EEQ de la numération des LT4, tout comme une soixantaine de laboratoire en Côte d'Ivoire grâce à un partenariat établi avec le QASI par le biais du coordonnateur local. L'objectif était de fournir au laboratoire une mesure de comparaison de ses résultats, de manière à lui assurer que le résultat obtenu ne diffère pas significativement de celui des autres laboratoires pour le même échantillon avec pour finalité la délivrance de résultats de grande précision et fiabilité [8]. De 2006 à 2015, le laboratoire d'immunologie et hématologie du CHU de Cocody avait eu un taux de participation de 83,33% à l'EEQ de la numération des LT4. Les sessions non effectuées coïncidaient avec la crise post-électorale de 2011 en Côte d'Ivoire. Cette période rendait difficile la coordination et l'acheminement des échantillons pour l'évaluation. Le taux de participation était tout de même satisfaisant et cela s'expliquait par plusieurs raisons notamment l'intérêt du laboratoire pour l'EEQ, la robustesse du cytomètre du laboratoire (FACSCalibur ^®^) qui avait connu très peu de pannes, la fourniture régulière des réactifs et le délai des sessions d'EEQ qui était long (environ deux mois), ce qui permettait au laboratoire de faire parvenir les résultats dans les délais requis.

L'évaluation de la performance des résultats de la numération des LT4 avait montré qu'il y'avait une bonne évolution au cours des sessions. Les valeurs SDI, autant pour le compte absolu que pour le compte relatif étaient en majorité inclus dans l'intervalle de confiance (-2; +2). Bergeron affirmait que la fréquence de participation à un programme de contrôle de qualité externe était le plus important facteur qui contribue à la réduction de l'ensemble des erreurs de phénotypage [9]. Le QASI avait opté pour un cycle d'expédition de 3 trois fois par an, contrairement aux programmes d'évaluation de la qualité dans les pays industrialisés qui adhèrent à un calendrier de six fois par année. La raison était que la plupart des participants au programme dans les pays en voie de développement n'avait pas accès à Internet pour la soumission en ligne des résultats de l'évaluation [7]. La performance des résultats du laboratoire pour la session initiale n'était pas satisfaisante. Il y'avait eu des erreurs de transcription, notamment en inversant les valeurs de deux échantillons de la session. L'EEQ est donc un processus très utile pour surveiller le bon déroulement de l'analyse. Des limites sont toutefois décrites par Hoeltge et al [10] qui affirment que l'EEQ ne permettait pas de mesurer l'intégralité du processus d'analyse au laboratoire, notamment dans la phase pré-analytique. Les SDI des sessions à compter des échantillons (QC_060, QC_061) oscillaient beaucoup plus dans l'intervalle de confiance et s'y écartaient par moment. Comme explications, des erreurs à la phase pré-analytique avaient été évoquées. Il s'agissait du long délai entre la réception des échantillons et leur analyse, source de rupture de la chaîne de froid ainsi que le remplacement du biotechnologiste de l'unité par un nouveau. Les variables qui pourraient influencer la fiabilité d'une analyse ont été décrites par des auteurs et sont généralement subdivisées en trois parties: les variables des phases pré-analytique, analytique et post-analytique [11]. Bonini P. et coll. [12] ont effectué une analyse des publications dans le domaine des erreurs de laboratoire et ont conclut que la grande partie des erreurs commises ont eu lieu dans les phases pré et post-analytiques. Ce constat est fait également par d'autres études [13, 14].

S'agissant de la précision des résultats rendus, ceux des échantillons de valeurs normales de CD4 étaient précis avec des CV compris entre les limites normales contrairement aux CV des échantillons contenant un faible taux de CD4 qui étaient au-delà des normes (>15%). Cet écart pour les échantillons de faible taux de CD4 pourrait s'expliquer par le fait que les préparations de CD4 de bas niveau sont moins stables et affichent une intensité de fluorescence diminuée [9]. Ce constat soulève le niveau de difficulté de l'analyse de cytométrie en flux de spécimens stabilisées qui contiennent des niveaux bas de cellules T-CD4. En plus, ces CV hors limites étaient beaucoup plus prononcés au début de l'évaluation et avaient tendance à se rapprocher de la limite des 15% au fil des sessions. Cette observation s'expliquait par l'inclusion à chaque envoi de nouveaux laboratoires nationaux dans le programme. En effet l'inclusion d'un nombre important de laboratoire lors d'une évaluation externe de la qualité contribue à élever le CV parce que ce sont des laboratoires débutants et inexpérimentés [9]. Globalement, les résultats rendus par le laboratoire d'immunologie et hématologie en matière de numération de CD4 étaient satisfaisants avec des taux de conformité de 89,58% pour le compte absolu et 91,87% pour le compte relatif. Malgré quelques insuffisances constatées, l'EEQ avait un impact positif sur la qualité de la numération de CD4 au laboratoire, contribuant ainsi à la qualité des prestations de soins.

## Conclusion

L'évaluation externe de la qualité (EEQ) est un outil indispensable qui permettait au laboratoire de surveiller la qualité de ses analyses. Toutefois des mesures correctives devraient être renforcées, particulièrement les visites de supervisions. Il est essentiel pour la qualité des soins que ce programme soit pérennisé, tout en envisageant la mise en place d'un programme national en sus pour augmenter le nombre de sessions d'évaluation du laboratoire par année.

### Etat des connaissances actuelle sur le sujet

- La détermination du taux de lymphocytes T-CD4 est essentielle au cours de l'infection à VIH;
- La cytométrie en flux pour le comptage des T-CD4 est usuelle dans les pays d'Afrique subsaharienne;
- L'évaluation externe de la qualité fournit une preuve objective de la qualité des analyses et indique les domaines dans lesquels une amélioration est nécessaire.

### Contribution de notre étude à la connaissance

- Montre l'importance de l'EEQ de la numération des lymphocytes T-CD4 au laboratoire d'immunologie et hématologie du CHU de cocody;
- Intérêt des mesures correctives continues dans le suivi-évaluation;
- Montre l'opportunité de la mise en place d'un programme d'EEQ élargi aux autres paramètres biologiques.

## Conflits d’intérêts

Les auteurs ne déclarent aucun conflit d'intérêts.
